# Epigenetics of gene expression in human hepatoma cells: expression profiling the response to inhibition of DNA methylation and histone deacetylation

**DOI:** 10.1186/1471-2164-7-181

**Published:** 2006-07-19

**Authors:** Luke O Dannenberg, Howard J Edenberg

**Affiliations:** 1 Department of Biochemistry and Molecular Biology, Indiana University School of Medicine, 635 Barnhill Drive, MS4063, Indianapolis, IN, USA

## Abstract

**Background:**

DNA methylation and histone deacetylation are epigenetic mechanisms that play major roles in eukaryotic gene regulation. We hypothesize that many genes in the human hepatoma cell line HepG2 are regulated by DNA methylation and histone deacetylation. Treatment with 5-aza-2'-deoxycytidine (5-aza-dC) to inhibit DNA methylation with and/or Trichostatin A (TSA) to inhibit histone deacetylation should allow us to identify genes that are regulated epigenetically in hepatoma cells.

**Results:**

5-aza-dC had a much larger effect on gene expression in HepG2 cells than did TSA, as measured using Affymetrix^® ^HG-U133 Plus 2.0 microarrays. The expression of 1504 probe sets was affected by 5-aza-dC (at p < 0.01), 535 probe sets by TSA, and 1929 probe sets by the combination of 5-aza-dC and TSA. 5-aza-dC treatment turned on the expression of 211 probe sets that were not detectably expressed in its absence. Expression of imprinted genes regulated by DNA methylation, such as *H19 *and *NNAT*, was turned on or greatly increased in response to 5-aza-dC. Genes involved in liver processes such as xenobiotic metabolism (*CYP3A4*, *CYP3A5*, and *CYP3A7*) and steroid biosynthesis (*CYP17A1 *and *CYP19A1*), and genes encoding CCAAT element-binding proteins (C/EBPα, C/EBPβ, and C/EBPγ) were affected by 5-aza-dC or the combination. Many of the genes that fall within these groups are also expressed in the developing fetal liver and adult liver. Quantitative real-time RT-PCR assays confirmed selected gene expression changes seen in microarray analyses.

**Conclusion:**

Epigenetics play a role in regulating the expression of several genes involved in essential liver processes such as xenobiotic metabolism and steroid biosynthesis in HepG2 cells. Many genes whose expression is normally silenced in these hepatoma cells were re-expressed by 5-aza-dC treatment. DNA methylation may be a factor in restricting the expression of fetal genes during liver development and in shutting down expression in hepatoma cells.

## Background

DNA methylation at the C5 position of a cytosine within a CpG dinucleotide is a primary mechanism in gene silencing [1]. Approximately 60% to 90% of the CpG dinucleotides in the genome of a vertebrate are methylated [2,3]. 5-aza-2-deoxycytidine (5-aza-dC) exerts its demethylating function by sequestering DNA methyltransferase 1 (DNMT1) to 5-aza-dC-substituted DNA via the irreversible binding of the cysteine in the catalytic domain of DNMT1 to the 6 position of the cytidine ring (4,5). This decreases the cellular concentration of DNMT1, which leads to genomic DNA demethylation in the course of successive cell divisions [6-8]. 5-aza-dC has been shown to reactivate silenced genes *in vitro *[9,10].

Regulation of gene expression in eukaryotes also involves the acetylation and deacetylation of histones, followed by chromatin remodeling [11-14]. Histones H3 and H4 are acetylated on the ε-amino groups of lysines to a greater extent than H2A or H2B [15]. Histone acetylation increases the accessibility of nucleosomal DNA to transcription factors and other regulatory molecules [16,17]. Turnover of the acetyl groups on histone molecules occurs very rapidly in cells [18,19] and the level of acetylation is controlled by an equilibrium of histone acetyltransferase and histone deacetylase (HDAC) activities [20-23]. Trichostatin A (TSA) is a reversible inhibitor of HDAC *in vitro *and *in vivo*, active at nanomolar concentrations, that causes a marked increase in histone acetylation [24].

DNA methylation and histone deacetylation can be mechanistically linked in gene silencing [25]. For example, the silenced rRNA genes can be re-expressed after either 5-aza-dC or TSA treatments [26-29] but treatment with both drugs together does not result in additional re-expression, suggesting that DNA methylation and histone deacetylation cooperate to silence rRNA gene expression [26]. Microarray analyses using human colorectal cancer cells showed that HDAC inhibitors alone could re-express some genes, but not genes that contain hypermethylated CpG islands; genes with hypermethylated CpG islands were only re-expressed after 5-aza-dC treatment [30]. 5-aza-dC and TSA have been shown to act in a synergistic fashion to re-express silenced genes in colorectal carcinoma cells [31,32].

The human hepatocellular carcinoma cell line HepG2 [33] synthesizes and secretes many of the major human plasma proteins, such as fibrinogen, plasminogen, and α2-macroglobulin [34]. These cells exhibit many cellular features of normal human hepatocytes [35] but also display characteristics resembling those of a fetal hepatocyte, because albumin and α-fetoprotein are both expressed; adult hepatocytes exclusively express albumin [36]. HepG2 cells also fail to express many of the hepatocyte-specific metabolic enzymes that characterize the neonatal and adult liver [34,37]. These cells express very low levels of the alcohol dehydrogenase 1B (*ADH1B*) and *ADH1C *genes, both of which are regulated by epigenetic mechanisms in HepG2 cells [38].

We were interested in determining what genes are regulated by DNA methylation and histone deacetylation in HepG2 cells. Affymetrix^® ^HG-U133 Plus 2.0 microarrays were used to measure the expression of approximately 54,000 transcripts in the presence and absence of 5-aza-dC and/or TSA. We report that the expression of many genes was altered upon 5-aza-dC treatment, and many fewer with TSA treatment. Groups of genes involved in xenobiotic metabolism, steroid biosynthesis, and CCAAT element binding were affected by 5-aza-dC (some exclusively with 5-aza-dC and TSA). Most of these genes are also expressed in the fetal and adult liver.

## Results

### Global gene expression in treated HepG2 cells

**Figure 1:**
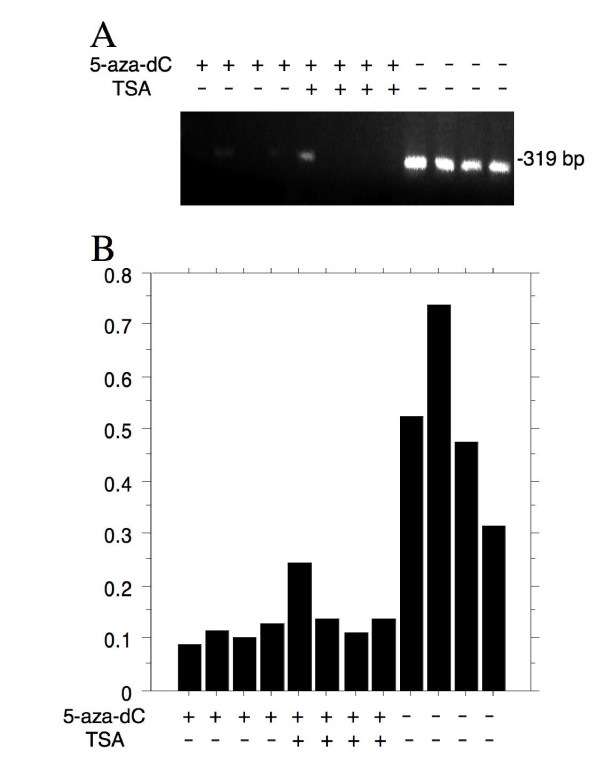
**Treatment of HepG2 cells with 2.5 μM 5-aza-dC results in DNA demethylation**. Demethylated DNA is cleaved by *Hpa II *digestion and cannot be amplified; therefore the amount of methylated DNA is reflected in the amount of PCR product. (A) Electropherogram of genomic DNA from untreated HepG2 cells or cells treated with 5-aza-dC; DNA was extracted and digested with *Rsa I *+ *Hpa II *and subjected to PCR using primers that flank the endogenously methylated *Hpa II *site at bp -2916 upstream of the *ADH1B *gene. (B) Quantitation of the PCR products produced in (A) using 2 X PicoGreen^® ^and an ABI PRISM^® ^7700 Sequence Detection System. The order of the quantitation is the same as the order of the PCR products seen in (A).

We treated HepG2 cells with 5-aza-dC to inhibit DNA methylation, TSA to inhibit histone deacetylation, or the combination of both 5-aza-dC and TSA, and measured changes in gene expression using Affymetrix^® ^HG-U133 Plus 2.0 microarrays. To ensure that 5-aza-dC treatments resulted in DNA demethylation, we performed methylation-sensitive PCR on a site 2916 bp upstream of the *ADH1B *translational start site. If the site is methylated, it is resistant to *Hpa II *digestion and can be amplified by PCR; if it is demethylated, this site will be cleaved and can no longer be amplified. Figure 1A demonstrates that genomic DNA from untreated cells was methylated at the bp -2916 *Hpa II *site, as demonstrated by the presence of robust 319 bp PCR products. DNAs from 5-aza-dC-treated HepG2 cells had methylation levels less than one-fifth that of untreated cells, as evidenced by the absence of PCR products (Figure 1A and 1B).

**Figure 2:**
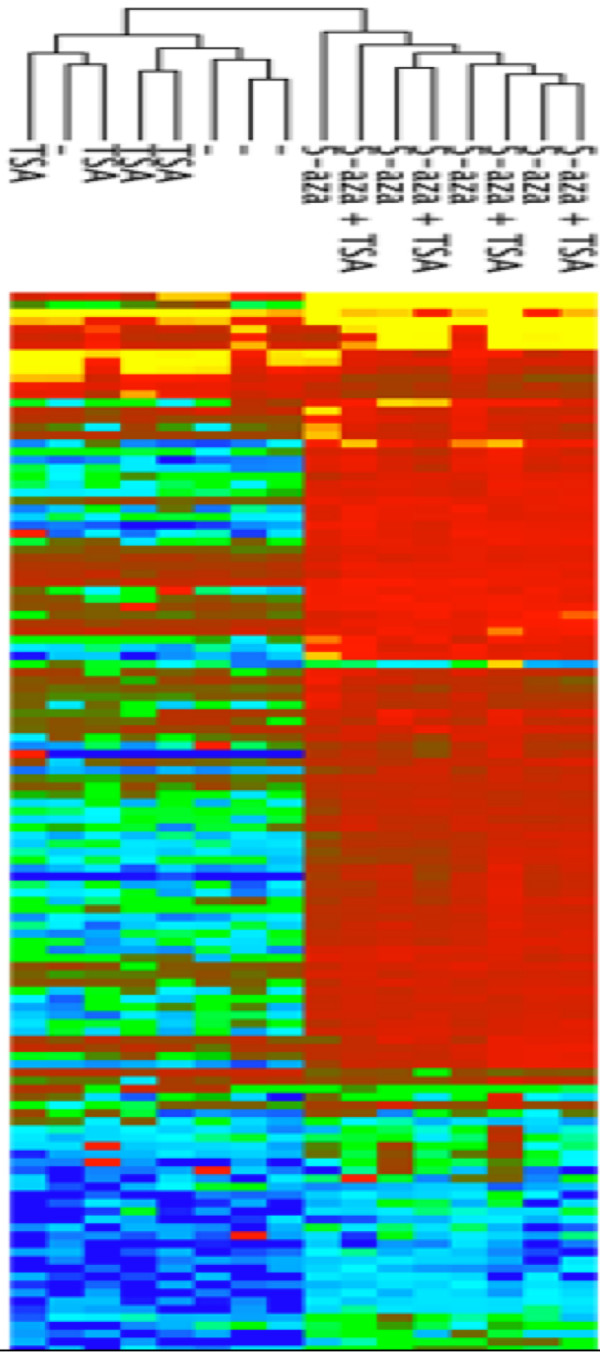
**Hierarchical cluster analysis of genes that vary across the experiment**. Genes that varied across the experiment (CV ≥ 0.6) were selected and clustered [75] based upon Euclidean distance. Colors represent differences from the median expression of the gene across different conditions: the top 5% are yellow, then in declining order from red to green (median), then progressively more blue (below median) to blue (bottom 5%).

Many genes were affected by the treatment of 5-aza-dC and/or TSA in HepG2 cells (Table 1). The expression of 1504 probe sets was changed by 5-aza-dC (p-value of 0.01; FDR ≤ 0.17), 535 by TSA (FDR ≤ 0.46), and 1929 by the combination of 5-aza-dC and TSA (FDR ≤ 0.13) (Table 1). Hierarchical clustering of the microarrays based on a set of 355 genes that varied most across the experiment (chosen based upon the ratio of standard deviation to the mean ≥ 0.6, without regard for whether the variation was within or between conditions) showed a clear separation of cells that received 5-aza-dC from cells that did not (Figure 2). Within this major division, cells treated with TSA did not segregate from untreated cells. Thus, 5-aza-dC had a marked effect on gene expression while TSA had only a minimal effect. At a p-value of 0.01, 5-aza-dC treatment increased the expression of 883 probe sets while decreasing the expression of 620 probe sets. TSA increased 244 and decreased 291 while 5-aza-dC plus TSA increased 1078 and decreased 851. Additional file 1 shows genes for which any of the treatments significantly altered its expression (p ≤ 0.05).

### Gene re-expression in 5-aza-dC treated cells

DNA methylation is known to play a large role in silencing the expression of many genes, thus we wanted to examine which genes in HepG2 cells were re-expressed by treatment with 5-aza-dC. We defined re-expressed stringently, requiring that the gene be undetectable ("Absent" as defined by the Affymetrix MAS5 algorithm) on any of the 4 arrays before treatment and detected ("Present") on all 4 arrays after treatment [see Additional file 2]. Treatment of HepG2 cells with 5-aza-dC restored the expression of 211 probe sets (188 different genes). Only two genes were turned off by 5-aza-dC. The additional presence of TSA did not substantially change the number of probe sets (208) that were re-expressed. A relaxed definition of re-expression (allowing one of the 8 arrays in the comparison to be called "Marginal") resulted in an additional 45 probe sets being re-expressed by 5-aza-dC and 33 by 5-aza-dC and TSA. Several genes regulated by imprinting were re-expressed or significantly up-regulated (*H19*) by treatment with 5-aza-dC, including *H19 *(maternally expressed), *NDN *(paternal), *NNAT *(paternal), and *MEG3 *(maternal) [see Additional file 2]. The latter three genes were not detectably expressed in untreated cells; *H19 *was minimally detected in untreated cells but is clearly being re-expressed. Another set of re-expressed genes included *TSPY1*, *DAZ2*, and *DAZ4*; these genes are involved in spermatogenesis [see Additional file 2].

Tissue culture and cancer cells are known to have an increase in genome wide methylation of CpG islands [39-42]. Thus, we examined the frequency of CpG islands within the promoters of 18 (10%, randomly selected) of the genes re-expressed by 5-aza-dC. Using CpG Island Finder [43,44] we found that 13 of 18 genes contained at least one CpG island. Of the 10 genes whose expression was confirmed by quantitative real-time RT-PCR (below) we found that 8 contained at least one CpG island.

Only four genes were turned on by the combination of 5-aza-dC plus TSA but remained off after treatment with 5-aza-dC or TSA alone (Table 2). The most striking of these was chromogranin A (*CHGA*), which is a central regulator of catecholamine storage and release [45]; expression was not detected on any of the arrays for 5-aza-dC, TSA, or untreated cells but it was present on all 4 arrays from 5-aza-dC plus TSA-treated cells. We confirmed this synergism by quantitative real-time RT-PCR. The expression of *CHGA *was not reliably detected in untreated cells or in two samples of TSA-treated cells. Expression was detected at very low-levels in two other TSA-treated samples and in all three 5-aza-dC-treated cells (*CHGA *C_t _- *GAPDH *C_t _[ddC_t_] = 7.7, corresponding to 0.5% of the level of *GAPDH*). Cells treated with the combination of both 5-aza-dC and TSA showed greatly enhanced expression of *CHGA*, approximately 80-fold, illustrating the synergy (ddC_t _= 1.4; 38% of the level of *GAPDH*).

### Groups of genes involved in liver processes are affected by 5-aza-dC

Genes affected by 5-aza-dC treatment were further analyzed to determine whether the expression of particular groups of genes (as defined by Gene Ontology) was affected by DNA methylation. We were initially interested in identifying groups of genes related to liver processes since this cell line is of liver origin (hepatoma). Table 3 shows groups of genes involved in xenobiotic metabolism, steroid biosynthesis, and CCAAT element binding that are regulated by DNA methylation; some genes are regulated by both DNA methylation and histone deacetylation, but not solely methylation. Many genes within these groups are also expressed in the developing fetal liver and adult liver (Table 3).

### Verification of microarray results by quantitative real-time RT-PCR

Quantitative real-time RT-PCR assays were performed to confirm microarray gene expression changes (Table 4). Genes were selected based on their role in the liver, imprinting status, cell cycle function, or as important transcription factors. The pattern of differences detected by RT-PCR was consistent with those from the microarrays, but the fold-changes obtained by real-time RT-PCR assays were, in most cases, much greater than fold-changes seen in microarray analyses. This observation was particularly true for genes re-expressed by 5-aza-dC treatment; in that case, the denominator in the microarray experiment (baseline, control value) is essentially zero (the gene is called "Absent") so the exact fold change calculated from microarray data is not meaningful or accurate. RT-PCR assays allow one to obtain a lower baseline value, and therefore a higher fold-change. It should be noted that we have not determined the amplification efficiency of each assay so these calculated fold-changes are based on 100% efficiency; this can overstate the fold change.

## Discussion

DNA methylation has a larger role in the inhibition of gene expression in the human hepatoma cell line HepG2 than does histone deacetylation. This is demonstrated by the greater effect by the DNMT inhibitor 5-aza-dC on gene expression than of the HDAC inhibitor TSA, as evidenced by the numbers of probe sets with altered gene expression (Table 1). It is also demonstrated by hierarchical clustering (Figure 2) in which the cells treated with 5-aza-dC, either alone or with TSA, segregated from cells not treated with TSA. 5-aza-dC treatment re-expressed 211 probe sets (188 genes) that had been silent in HepG2 cells [see Additional file 2], and up regulated more genes than were down regulated. In the overall pattern of gene expression, we do not detect much synergy between 5-aza-dC and TSA on gene expression in these cells. A small number of genes (4) required both 5-aza-dC and TSA for re-expression; we confirmed the synergistic effects of 5-aza-dC and TSA on *CHGA *gene expression by performing quantitative real-time RT-PCR.

A subset of autosomal genes, known as imprinted genes, display expression exclusively from one of the parental alleles [46]. The imprinted genes *NDN*, *NNAT*, and *MEG3 *were turned on in 5-aza-dC-treated cells (Table 4 and see Additional file 2), demonstrating that the DNA demethylation was sufficient to re-express genes silenced by DNA methylation in the HepG2 cells. The expression of *H19 *(maternal) was greatly increased (156-fold [see Additional file 2]; essentially turned on) while the expression of *IGF2 *(paternal) was decreased (-4.2-fold, p = 0.02 [see Additional file 1]. Both genes lie together on chromosome 11p15, and the upstream region of *H19 *is the downstream region of *IGF2*; this intergenic region, also known as the differentially methylated region, is responsible for monoallelic expression of both genes [47-49]. If this DMR is unmethylated, the transcription factor CTCF can bind and block the activity of an enhancer that activates *IGF2 *expression; this allows the enhancer to now interact with the *H19 *promoter [47]. Our results are consistent with this.

5-aza-dC also turned on the expression of several Y chromosome-specific genes [see Additional file 2]. Past studies using prostate cancer cell lines showed that DNA methylation regulates the expression of these genes and that 5-aza-dC can restore their expression [50]. The expression of *TSPY1 *is normally restricted to the testis where it plays a role in spermatogonial proliferation [51]. *TSPY1 *contains a putative CpG island in its promoter that is hypermethylated in melanoma cell lines; treatment with 5-aza-dC can restore *TSPY1 *expression [52]. The *DAZ *cluster of genes are thought to be involved in germ cell differentiation in humans and old world monkeys [53]; deletions in this region can cause infertility in males [54]. This finding is interesting given that we treated hepatoma cells, a cell type that normally does not express spermatogenesis genes. It confirms that DNA methylation plays a large role in shutting down the expression of many genes that have no role in contributing to the proper functioning of that cell type.

We further analyzed genes that were significantly affected by 5-aza-dC treatment and identified groups of genes that are involved in xenobiotic metabolism, steroid biosynthesis, and CCAAT element binding (Table 3). The most affected in the xenobiotic metabolism group, the *CYP3A *genes, play major roles in metabolic disposition of wide variety of drugs [55]. *CYP3A4 *and *CYP3A5 *comprise nearly 30%-40% of total hepatic cytochrome p450 [55] while *CYP3A7 *expression is restricted to the fetal liver [56]. *CYP3A *expression in untreated HepG2 cells is fairly low (signals ranging from 370 to 968), suggesting that their expression is reduced in these partially de-differentiated cells; however, 5-aza-dC greatly increased the expression of each *CYP3A *gene. This increase in expression was confirmed by real-time RT-PCR for *CYP3A7 *(Table 4).

Genes within the steroid biosynthesis group also included two *CYP *genes. The *CYP19A1 *gene, which was re-expressed by 5-aza-dC treatment [see Additional file 2], encodes the main estrogen biosynthesis enzyme, which converts androgen to estrogen [57]. *CYP19A1 *expression can be increased in endometriotic stromal cells upon *in vivo *down-regulation of C/EBPβ [58]. This finding is interesting given that C/EBPβ expression was decreased by 2-fold; this may contribute to *CYP19A1 *re-expression. The CYP17A1 enzyme (Table 3) functions in the biosynthesis of testosterone through the regulation of 17-hydroxylase and 17, 20-lyase [59]. CYP17A1 is not expressed in the fetal or adult liver; hence DNA methylation may be a major mechanism to transcriptionally silence its expression in the liver.

We examined seven genes that bind CCAAT DNA elements to regulate transcription of many important liver genes (Table 3). Most notable are the three genes encoding C/EBPα, C/EBPβ and C/EBPγ (Table 3; *CEBPA*, *CEBPB*, and *CEBPG*) that were differentially affected by 5-aza-dC or TSA and the combination of 5-aza-dC and TSA. Surprisingly, the expression of *CEBPB *and *CEBPG *were decreased by 5-aza-dC; because methylation generally inhibits gene expression [1], this down-regulation could be a secondary effect of the increase in an inhibitory factor. *CEBPA *was up-regulated by TSA and the combination of 5-aza-dC and TSA, but not with 5-aza-dC alone. The transcriptional state of many other hepatocyte-specific genes in these cells is likely altered based on the expression profile of these C/EBP genes. *CEBPB *can generate two isoforms from a single mRNA: the full-length protein termed liver activation protein (LAP) or the truncated liver inhibitory protein (LIP) isoform [60-63]. LIP is a dominant-negative factor that can attenuate transcription by forming a heterodimer with LAP to negate the transactivation ability of LAP [62]. LIP is still able to attenuate the transcriptional activity of LAP even when present at sub-stoichiometric amounts (i.e. LAP/LIP ratio of 5:1) [62,64]. C/EBPγ heterodimerization with C/EBPα and LAP can also attenuate transcription of C/EBP responsive promoters, suggesting dominant negative regulation by this factor [65]. Hence, the decreased expression of C/EBPβ and C/EBPγ transcripts may aid in increasing the expression of the hepatocyte-specific genes they regulate.

The *CEBPA *gene is regulated by histone deacetylation (and slightly increased with 5-aza-dC and TSA) in the HepG2 human hepatoma cell line. An increase in *C*/EBPα expression will increase the expression of several hepatocyte-specific genes that are regulated by this factor. However, a larger picture emerges from our data. HepG2 cells display many characteristics of fetal and adult hepatocytes. Many genes regulated by DNA methylation that function in liver processes (Table 3) are expressed in the fetal and adult liver. DNA demethylation may be, in part, responsible for silencing the expression of fetal liver genes. C/EBPα and C/EBPβ may play important roles in this. The haptoglobin (Hp) gene, which is important in liver development, is transcriptionally regulated by C/EBPα and C/EBPβ in the rat liver; Dinic *et al*. have shown that Hp transcription is regulated by C/EBPα during normal liver development while C/EBPβ regulates this gene during the acute phase response during the later phase of differentiation and in the adult [66]. Also, the suppression of *C/EBPα *expression may be a prerequisite to biliary cell differentiation in a hepatoblast population in the developing mouse liver [67].

## Conclusion

In summary, we demonstrate that DNA methylation plays a much larger role than histone deacetylation in regulating gene expression in HepG2 human hepatoma cells. Many genes were in fact turned on in these cells by the DNMT inhibitor 5-aza-dC; which included imprinted genes and spermatogenesis genes. 5-aza-dC affected groups of genes involved in liver processes such as xenobiotic metabolism, steroid biosynthesis, and CCAAT element binding. A majority of the genes in these groups are expressed in the developing fetal and adult liver indicating that DNA methylation may play a role in restricting the expression of fetal liver genes and in shutting down expression in hepatoma cells.

## Methods

### Drug treatments

5-aza-dC (Sigma, St. Louis, MO) and TSA (Sigma) were prepared in dimethyl sulfoxide (DMSO) (Sigma). HepG2 cells (HB-8065; ATCC, Manassas, VA) (5 × 10^5 ^cells) were plated on 100 mm culture dishes and grown in minimum essential medium (Sigma) with 10% fetal bovine serum (Life Technologies, Rockville, MD) and 2 mM glutamine in a 5% CO_2 _atmosphere. A 2 × 2 experimental design was used: cells were treated with 2.5 μM 5-aza-dC, 500 nM TSA, both 2.5 μM 5-aza-dC and 500 nM TSA, or DMSO (untreated). Plates were seeded and harvested at the same time; appropriate dishes were treated with 5-aza-dC 48 h after cell seeding and their media and drugs were replaced every 24 h. TSA was incubated with appropriate dishes for the last 24 h before harvesting. DNA and RNA were extracted 4 days after the initial 5-aza-dC treatment using TRIzol^® ^(Invitrogen, Carlsbad, CA).

### DNA methylation analysis

Methylation-sensitive PCR was modified from a protocol [68] and quantitation of PCR products were previously described by Dannenberg *et al*. [38]. This assay is based on the fact that *Hpa II *cannot digest a methylated CCGG site; thus fragments containing methylated CCGG sites remain intact and can be amplified by PCR, whereas unmethylated CCGG sites are cut and cannot be amplified. The region upstream of *ADH1B *from bp -2956 to -2638 was analyzed using primers HE1741/1742 (Table 5).

### Microarray analysis

Total RNA prepared from HepG2 cells was treated with 1 U of DNase I, RNase-Free (Roche, Indianapolis, IN) per microgram of RNA. An RNeasy^® ^Mini Kit (Qiagen, Valencia, CA) was used to purify RNA. RNA quality was then tested using a Bioanalyzer (Agilent Technologies, Palo Alto, CA) and by measuring absorbance from 210 to 350 nm. RNA preparations from four independent dishes from each experimental condition (5-aza-dC, TSA, both 5-aza-dC and TSA, and untreated) were separately processed and analyzed. Ten micrograms of total RNA was synthesized into cDNA for each sample, and then biotinylated cRNA was generated by *in vitro *transcription, following the standard Affymetrix protocols [69]. Biotinylated cRNAs were fragmented, and each sample was hybridized to an Affymetrix^® ^HG-U133 Plus 2.0 gene chip (54,675 probe sets) at 42°C for 17 h. Chips were washed, stained, and scanned following the standard Affymetrix protocol [69].

### Statistical analysis

Expression data generated by the Affymetrix Microarray Suite^®^, version 5.0, were exported and analyzed using Microarray Data Portal (MDP) [70]. Data have been deposited into GEO [71] under Series accession number GSE5230. Probe sets that were not called present on at least 50% of the arrays in at least one-treatment group were eliminated before further analysis; these primarily represent noise [72,73]. Log-transformed signals were then compared using Welch's approximate *t*-test, which allows for unequal variance. False discovery rate (FDR) was calculated according to Benjamini and Hochberg [74]. Genes were considered turned from "off to on" or "on to off" if the difference of the fraction present was +/- 1; that is, if genes went from "Absent" (according to the MAS5 algorithm) in all 4 arrays to "Present" in all four arrays, or *vice versa *and the log signal differed significantly (p ≤ 0.05). Genes turned on by the combination of 5-aza-dC plus TSA (as defined above) but not by 5-aza-dC alone were also selected, based on a p value for both vs. 5-aza-dC <0.05 and difference in fraction present ≥ 0.5. Gene groupings were created using Gene Ontology on MDP; the CCAAT-binding protein genes were grouped manually. Fetal liver expression was determined by using the Expression Profile Viewer (NCBI). Genes are considered as expressed in the fetal liver if they are expressed in the liver and also expressed in the embryo.

Putative CpG islands were identified using the CpG Island Finder [43,44]. We selected a random 10% of the genes that were re-expressed [see Additional file 2] and examined their promoters from -1500 to +500 bp. We also examined 10 genes that were confirmed by RT-PCR (Table 4). CpG Island Finder uses a sliding window 201 bp in length to calculate the CpG dinucleotide percentage for each window where it defines the maximum of these CpG percentages as the CpG score and the corresponding window as the CpG window [43]. Assuming a bimodal normal distribution and an overlap of 10% (5% from each group), 6.5 as a cutoff value is often used because it satisfies the condition μ+2σ < 6.5 < μ-2σ [43]. However, we used the default value of 6%, set by the CpG Island Finder [44], to identify promoter CpG islands.

### Hierarchical clustering

A set of 355 genes that varied across the experiment (without consideration of whether variation was within or between conditions, to avoid bias) was selected based upon a coefficient of variation (CV; ratio of standard deviation to mean) of 0.6 or greater. Hierarchical clustering of the microarrays [75] was performed using MDP.

### Real-time quantitative RT-PCR

The following human mRNA sequences: *CDK10, CEBPA, CEBPB, CHGA, CYP3A7, GSTP1, H19, NNAT, PKIB, S100A4*, and *TSPY1 *were used to design gene-specific primers for real-time RT-PCR using MacVector (Accelrys, San Diego, CA). Primer sequences were tested for uniqueness by aligning them against the human genome using NCBI BLAST [76]. The RT-PCR products and Accession numbers (GenBank) for each mRNA sequence are listed in Table 6; the sequences of the primers used are listed in Table 5.

Four 500 ng aliquots of TSA-treated and untreated RNA preparations used in the microarray analyses and three 500 ng aliquots of 5-aza-dC-treated and 5-aza-dC and TSA-treated (only three used for these conditions due to little or no total RNA available after microarray analyses) were reverse transcribed using the SuperScript First Strand Synthesis System (Invitrogen). Resulting cDNAs were diluted 1:25, then amplified using SYBR Green PCR Master Mix (Applied Biosystems, Foster City, CA). *GAPDH *(glyceraldehyde-3-phosphate dehydrogenase) expression was quantified using TaqMan^® ^GAPDH Control Reagents (Applied Biosystems) as an internal control to normalize each gene against (note: *GAPDH *amplification not run in parallel with each gene due to limiting amounts of cDNA). PCR products were quantified in real-time using an ABI PRISM^® ^7700 Sequence Detection System (Applied Biosystems). PCR conditions were 50°C for 2 min, 95°C for 10 min, 40 cycles of 95°C for 15 sec and 51–60°C for 20 sec. C_t _values for each gene and condition were normalized against *GAPDH *and then each condition was normalized against the untreated cells to obtain a fold-change. Welch's *t*-tests were performed to analyze the real-time RT-PCR data. We did not determine the amplification efficiency of each assay; the fold-changes are calculated based on 100% efficiency.

## Authors' contributions

LOD performed the experiments, data analysis, and drafted the manuscript. HJE guided the experiments, performed data analysis, and participated in the writing of the manuscript.

## Supplementary Material

Additional File 1 **Genes that differed between untreated and treated cells**. Genes that were present in at least half of the arrays in at least one group, and that significantly differ (p ≤ 0.05) between untreated cells and any of the treatments (5-aza-dC, TSA, or both 5-aza-dC and TSA). Genes are sorted in the order: gene name, Unigene number, and condition; genes lacking gene symbols are sorted in order of Unigene number. Click here for file

Additional File 2 **Probe sets re-expressed by 5-aza-dC**. Probe sets that were re-expressed by 5-aza-dC treatment are shown (see Methods for details). Click here for file

## Figures and Tables

**Table 1:** Number of probe sets changed by different treatments

**p-/values**^**1**^	**5-aza-dC**	**TSA**	**5-aza-dC and TSA**
**0.05**	3974 (.326)^2^	2161 (.569)	4927 (.257)
**0.01**	1504 (.172)	535 (.460)	1929 (.132)
**0.001**	346 (.075)	55 (.455)	468 (.053)
**0.0001**	73 (.041)	10 (.200)	126 (.024)

^1^Based on Welch's *t*-test log(signal).

^2^Number and (FDR) of probe sets significant at each p-value.

**Table 2:** Genes re-expressed by 5-aza-dC and TSA, but not by either drug alone

Gene Symbol	Unigene	Affymetrix ID	Fold Change	p-value	Control Signal	5-aza-dC + TSA Signal	5-aza-dC Signal	Description
CHGA	Hs.150793	204697_s_at	3.03	0.012	214	648	214	Chromogranin A (parathyroid secretory protein 1)
TKTL1	Hs.102866	214183_s_at	57.92	0.00004	49	2809	1276	Transketolase-like 1
BG1	Hs.250616	206465_at	6.16	0.00007	172	1057	630	Lipidosin
-	Hs.399852	1559213_at	43.98	0.0003	19	842	193	IMAGE:5394246, mRNA

**Table 3:** Groups of genes involved in liver processes that are affected by 5-aza-dC.

**Gene Symbol**	**Unigene**	**Affymetrix ID**	**Fold Change**	**p-value**	**FDR**	**Control Signal**	**5-aza-dC Signal**	**Description**
**Xenobiotic Metabolism**								
ABHD6^	Hs.476454	221552_at	1.82	0.00394	0.12238	634	1151	Abhydrolase domain containing 6
AKR1C1^	Hs.460260	1555854_at	2.92	0.00027	0.04452	345	1007	Aldo-keto reductase family 1, member C2 (dihydrodiol dehydrogenase 2; bile acid binding protein; 3-a
CYP3A4^	Hs.442527	205999_x_at	1.78	0.01792	0.21785	370	660	Cytochrome P450, family 3, subfamily A, polypeptide 4
CYP3A5^	Hs.150276	214234_s_at	2.53	0.00159	0.08806	968	2447	Cytochrome P450, family 3, subfamily A, polypeptide 5
CYP3A7^	Hs.111944	211843_x_at	4.11	0.00387	0.12175	515	2120	Cytochrome P450, family 3, subfamily A, polypeptide 7
HAMP^	Hs.8821	220491_at	2.34	0.01066	0.17693	1076	2512	Hepcidin antimicrobial peptide
UGT1A6^	Hs.546397	215125_s_at	2.73	0.00318	0.11285	143	389	UDP glycosyltransferase 1 family, polypeptide A9
UGT2B15^	Hs.150207	207392_x_at	1.79	0.01965	0.22477	323	577	UDP glycosyltransferase 2 family, polypeptide B15
UGT2B28	Hs.137585	211682_x_at	-2.69	0.00131	0.08111	11036	4109	UDP glycosyltransferase 2 family, polypeptide B28
**Steroid Biosynthesis**								
CYP17A1	Hs.438016	205502_at	1.92	0.00208	0.09647	325	622	Cytochrome P450, family 17, subfamily A, polypeptide 1
CYP19A1^	Hs.511367	203475_at	2.43	0.00356	0.11831	470	1144	Cytochrome P450, family 19, subfamily A, polypeptide 1
CYP51A1^	Hs.417077	202314_at	-1.31	0.00975	0.17073	11672	8910	Cytochrome P450, family 51, subfamily A, polypeptide 1
DHCR7^	Hs.503134	201791_s_at	-1.4	0.02202	0.23665	3628	2588	7-dehydrocholesterol reductase
FDFT1^	Hs.546253	208647_at	-1.37	0.00820	0.16076	24377	17858	Farnesyl-diphosphate farnesyltransferase 1
FDXR^	Hs.69745	207813_s_at	2.31	0.00046	0.05618	2066	4764	Ferredoxin reductase
HMGCR^	Hs.11899	202539_s_at	-1.34	0.03567	0.28427	10570	7864	3-hydroxy-3-methylglutaryl-Coenzyme A reductase
HSD17B1^	Hs.500159	205829_at	6.02	0.00853	0.16330	157	944	Hydroxysteroid (17-beta) dehydrogenase 1
HSD17B2^	Hs.162795	204818_at	2.68	0.00198	0.09502	1818	4869	Hydroxysteroid (17-beta) dehydrogenase 2
LSS^	Hs.517366	202245_at	-1.32	0.00717	0.15151	5751	4356	Lanosterol synthase (2,3-oxidosqualene-lanosterol cyclase)
PMVK	Hs.30954	203515_s_at	1.6	0.00661	0.14527	1046	1674	Phosphomevalonate kinase
RODH^	Hs.524513	37512_at	3.67	0.00046	0.05648	173	634	3-hydroxysteroid epimerase
SC5DL^	Hs.287749	211423_s_at	-1.19	0.01127	0.18081	10122	8494	Sterol-C5-desaturase (ERG3 delta-5-desaturase homolog, fungal)-like
SQLE^	Hs.71465	209218_at	-1.32	0.00603	0.14008	16131	12229	Squalene epoxidase
STARD4^	Hs.93842	226390_at	-1.22	0.01833	0.21953	4385	3593	START domain containing 4, sterol regulated
								
**CCAAT element binding proteins**								
CEBPA*^	Hs.76171	204039_at	1.69	0.00049	0.04052	3106	5262	CCAAT/enhancer binding protein (C/EBP), alpha
CEBPB^	Hs.517106	212501_at	-1.95	0.01864	0.22082	28789	14762	CCAAT/enhancer binding protein (C/EBP), beta
CEBPG^	Hs.429666	225527_at	-2.15	0.04308	0.30614	3734	1741	CCAAT/enhancer binding protein (C/EBP), gamma
CEBPZ*^	Hs.135406	203341_at	-1.34	0.00286	0.08197	4880	3655	CCAAT/enhancer binding protein zeta
CUTL1*^	Hs.438974	214743_at	-1.57	0.00957	0.1289	1672	1067	Cut-like 1, CCAAT displacement protein (Drosophila)
NFIC*^	Hs.170131	226895_at	1.23	0.01727	0.16248	1452	1786	Nuclear factor I/C (CCAAT-binding transcription factor)
NFYC^	Hs.233458	202215_s_at	1.38	0.01163	0.18301	1612	2228	Nuclear transcription factor Y, gamma

* = Genes that are only significantly affected by both 5-aza-dC and TSA.

^ = Expressed in fetal and/or adult liver.

**Table 4:** Validation of fold-changes from microarray analyses by real-time RT-PCR

		**5-aza-dC**		**TSA**		**5-aza-dC and TSA**	
	**Control**	**Array**^**1**^	**RT-PCR**^**1**^	**Array**	**RT-PCR**	**Array**	**RT-PCR**
***CDK10***^**3**^	300*	3.5 (.00013)	8.3 (.00000)	NT^2^	NT	3.8 (.00001)	7.2 (.00000)
***CEBPA***^**3**^	3106	NT	NT	1.43 (.0038)	4.16 (.004)	1.7 (.00049)	10.4 (.00000)
***CEBPB***^**3**^	28789	-2.0 (.019)	-2.6 (.0008)	NT	NT	-2.0 (.019)	-3.5 (.00009)
***CYP3A7***^**3**^	515*	4.0 (.0043)	11 (.00000)	NT	NT	4.3 (.0043)	8.0 (.00000)
***GSTP1***^**3**^	367**	8.5 (.00014)	18200 (.00000)	NT	NT	12 (.00005)	107000 (.00000)
***H19***^**3**^	255	156 (.00000)	5080 (.00000)	NT	NT	141 (.00000)	330 (.00000)
***NNAT***	205*	8.0 (.0073)	38 (.00000)	NT	NT	10.8 (.0053)	33 (.00000)
***PKIB***	464*	7.4 (.0029)	70 (.00000)	NT	NT	6.6 (.0013)	35 (.00000)
***S100A4***^**3**^	281*	8.8 (.0012)	27 (.00000)	3.0 (.00039)	4.9 (.00000)	8.2 (.00018)	11 (.00000)
***TSPY1***	11*	96 (.00002)	532 (.00000)	NT	NT	153 (.00000)	1290 (.00000)

*: Called "Absent" on all 4 control arrays; **: called "Absent" on 3 of 4 control arrays

^1^The fold-change for either the microarray analyses or real-time RT-PCR assays (relative fold-change). P-values (Welch's *t*-test) are shown in parentheses.

^2^NT = not tested, because it was not significantly affected in the microarray analyses.

^3^Expression is observed in the fetal liver.

**Table 5:** Primer sequences

**Primers**	**Sequences**
HE1741	CCAGGGATTAGGAGTGGACC
HE1742	GGAGGGGAAGAGCAGTTGTC
HE2251	GATTGGACCTCATTACATAAGGG
HE2252	TCTTTTGGGGTTAGCAAGTTG
HE2261	AGGGTTGTGTGAAGACAGCG
HE2262	TTGTTGCGGTGGTGGGGAAAG
HE2245	GGACCCTCAGCCTTGTTTG
HE2246	AACCCCCCTCACCTCATTGG
HE2255	TGTCCAAACCAACCGCAC
HE2256	AACAGCAACAAGCCCGTAG
HE2253	CCTGATGTCCAGCAGAAAGTGC
HE2254	GTTAGAAGAAGTCCTCCAAAGCG
HE2219	TACCAGTCCAATACCATCCTGCG
HE2220	TGCCTTCACATAGTCATCCTTGC
HE2241	ACTGGTTGGAGTTGTGGAGACG
HE2242	TGGAATGCTTGAAGGCTGCTC
HE2369	AATCAAAACACCGCACCAG
HE2370	ATCAGTGAGGGGCAAGGGGGGTTC
HE2243	CAGACGGAACCTCAGATTTGCC
HE2244	CAGTGCGATTTGGAAGTAACCTG
HE2265	GCAAAGAGGGTGACAAGTTCAAGC
HE2266	GCCAGGGTGGAAAAAAAAAAGTGC
HE2249	CTCTGACCACAACTTCGCAG
HE2250	CCACATTTACCCCCTCTTCC
HE3106	CTACCCCGAGGAGAAGAAAGAG
HE3107	CTGTGTTCAGAGAAGGAATCGTC

**Table 6:** Real-time RT-PCR products. Positions are relative to the translational start site.

**Gene**	**Accession_No.**	**Position**	**Primer pair**
*CDK10*	NM_003674.2	+436 to +946	HE2261/62
*CEBPA*	NM_004364.2	+1900 to +2254	HE2245/46
*CEBPB*	NM_005194.2	+1216 to +1483	HE2255/56
*CHGA*	NM_001275	+1629 to +2146	HE3106/07
*CYP3A7*	NM_000765.2	+972 to +1453	HE2253/54
*GSTP1*	NM_000852.2	+189 to +365	HE2219/20
*H19*	BC_053637	+1342 to +1546	HE2241/42
*NNAT*	NM_005386	+613 to +967	HE2369/70
*PKIB*	NM_181795.1	+115 to +454	HE2243/44
*S100A4*	NM_002961	+61 to +387	HE2265/66
*TSPY1*	NM_003308.2	+782 to +1080	HE2249/50
